# Supplementary material for: Epstein–Barr Virus Infection Is Associated with Elevated Hepcidin Levels
Source: Int J Mol Sci. 2023 Jan 13;24(2):1630. doi: 10.3390/ijms24021630 (PMC9862144; doi:10.3390/ijms24021630)
Supplement: Supplementary file 1 [file ijms-24-01630-s001.zip › ijms-2046717-SI.pdf]

International Journal of *Molecular Sciences*

Article

Epstein-Barr Virus and *Helicobacter pylori* Coinfection is Associated with Elevated Hepcidin Levels

Ximena Duque [1], Eugenia Mendoza [1], Segundo Morán [2], Mayra C. Suárez-Arriaga [3], Abigail Morales-Sánchez [3], José I. Fontes-Lemus [3], Diana A. Domínguez-Martínez [3] and Ezequiel M. Fuentes-Pananá [3*]

Supplementary Table S1. Associations between nutritional and EBV infection status in children

**Infection**	**EBV pos** n=131		**EBV neg** n=12		**p value**[a]
	**n**	**%**	**n**	**%**	
**Body mass index**					
Normal	84	64.1	10	83.3	
Overweight/Obesity	47	35.9	2	16.7	0.220
**Height to age**					
Normal	123	93.9	12	100.0	
Stunting	8	6.1	0	0.0	1.000
**Iron Status**					
Normal	110	84.0	10	83.3	
Iron deficiency	20	15.3	2	16.7	
Non-iron deficiency anemia	1	0.7	0	0.0	1.000
	H. pylori **or EBV or inflammatory process +** n=133		***H. pylori*** **and EBV and inflammatory process -** n=7		
**Body mass index**					
Normal	88	65.2	6	85.7	
Overweight/Obesity	47	34.8	1	14.3	0.422
**Height to age**					
Normal	125	94.0	7	100.0	
Stunting	8	6.0	0	0.0	1.000
**Iron Status**					
Normal	111	83.5	7	100.0	
Iron deficiency	21	15.8	0	0.0	
Non-iron deficiency anemia	1	0.7	0	0.0	0.615

[a] Fisher´s exact test

Supplementary Table S2. Genes used for the bioinformatic analysis of hepcidin expression regulation.

Gene	Reference
Hepcidin (*HAMP*)	[99]
Ferroportin 1 (*SLC40A1*)	[99]
Hemojuvelin (*HJV*)	[99]
Divalent metal transporter 1 (*DMT1*)	[99]
Iron-regulatory protein 1 (*IRP-1*)	[99]
Iron-regulatory protein 2 (*IRP-2*)	[99]
Hypoxia-inducible factor (*HIF1A*)	[99]
Transferrin receptor 1 (*TFRC*)	[99]
Transferrin receptor 2 (*TFR2*)	[99]
Homeostatic iron regulator protein (*HFE*)	[99]
Bone morphogenetic protein 6 (*BMP6*)	[99]
Bone morphogenetic protein receptor II (*BMPRII*)	[99]
SMAD family member 4 (*SMAD4*)	[99]
Matriptase 2 (*TMPRSS6*)	[99]
Transmembrane epithelial antigen of the prostate protein 1 (*STEAP1*)	[100]
Transmembrane epithelial antigen of the prostate protein 1B (*STEAP1B*)	[100]
Transmembrane epithelial antigen of the prostate protein 2 (*STEAP2*)	[100]
Transmembrane epithelial antigen of the prostate protein 3 (*STEAP3*)	[100]
Transmembrane epithelial antigen of the prostate protein 4 (*STEAP4*)	[100]
Heme carrier protein 1 transporter (*HCP1*)	[100]

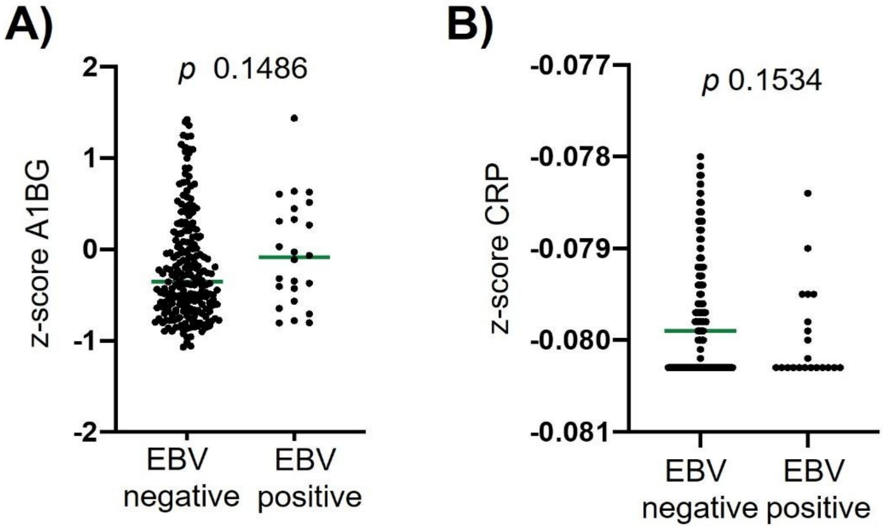

Supplementary Figure S1. EBV infection does not associate with the expression of acute phase proteins in gastric cancer patients. (A) *A1BG* and (B) *CRP* gene expression was compared between EBVaGC and EBVnGC patients. Outliers were identified using the ROUT analysis. Mann-Whitney test.

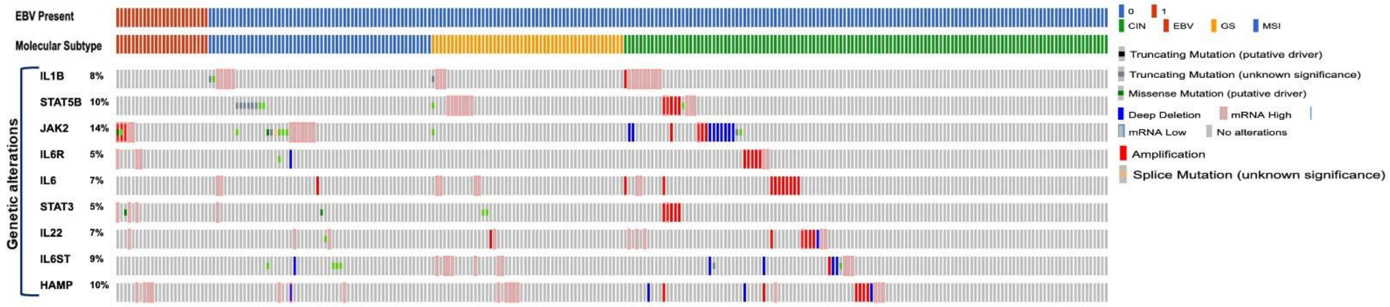

Supplementary Figure S2. The *HAMP* upregulated expression in EBVaGC is not explained by an increased rate of mutations in regulatory genes. Heatmap shows single nucleotide variants and copy number aberrations in genes related to *HAMP* expression in the hepcidin-regulatory inflammatory pathway.
